# Effects of Urban Producer Service Industry Agglomeration on Export Technological Complexity of Manufacturing in China

**DOI:** 10.3390/e22101108

**Published:** 2020-09-30

**Authors:** Xinyu Gao, Chengpeng Lu, Jinhuang Mao

**Affiliations:** Institute of County Economic Development & Rural Revitalization Strategy, Lanzhou University, Lanzhou 730000, China; xygao@lzu.edu.cn (X.G.); maojh@lzu.edu.cn (J.M.)

**Keywords:** producer service industry, manufacturing, export technological complexity, regional heterogeneity, China

## Abstract

Based on the measurement of producer service industry agglomeration and export technological complexity of manufactured products in 288 Chinese cities from 2000 to 2015, this paper illustrates the evolvement and spatial characteristics of the two factors through visualization figures, and discusses the effects of producer services agglomeration on export technological complexity of manufacturing through robust panel data models. The findings are as follows: as with the influence of industrial connection, empirical outcomes indicate that urban producer service agglomeration can promote technological complexity of export manufacturing on the full-sample level. Visualization analysis shows that the scale of producer service industry agglomeration and the export technological complexity of manufactured products around Chinese cities kept rising constantly during the study period. However, although the export technological complexity displayed a trickle-down effect, the producer service industry agglomeration experienced continuous polarization both on the national and the regional levels. Accordingly, as is shown in the empirical analysis by areas, regions with strong support from producer service industry saw a remarkable promotion in the export manufacturing technology, while the northwest and the northeast gradually lagged behind. Such results sufficiently prove that heterogeneity does exist in the performances of industrial connection between producer service industry and export manufacturing in cities of different regions in China.

## 1. Introduction

With the rapid improvement of productivity and the continuous optimization of industrial structure since the 1960s, the developed countries have undergone a trend of economic structure transformation from “industrial” to “service-oriented”, mainly due to the outstanding contribution that the producer service industry has made to economic growth [1,2]. Running through almost all the intermediate processes of production, producer services are conductive to promoting sector transactions, lowering costs, and deepening professional division of production on industrial chains, and therefore accelerating the upgrade of value chains [3]. On the spatial distribution of producer services enterprises, a great many of researches have demonstrated that producer services show a “natural tendency to cluster” [4] in pursuit of externalities like technological spillover [5], professional communication [6], stable customer base [7], concentration of educated labor force and its “labor pool” effect [8], which form the localized advantages making producer service clusters highlands of innovation [9]. In the process of producer services agglomeration, there gradually establishes a well-developed local network for factor transaction and the information exchange [10], where the speed of updating technology [11], ties of enterprise cooperation [12] and so on strengthen the development of producer service industry in turn. Thus, it is rational that the larger the scale of producer services agglomeration, the more maturely the local producer service industry develops [6].

Coffey (2000) [13] defined producer services as a knowledge-intensive industry that provides necessary professional intermediate services for manufacturing. Mutually influencing and depending closely on each other, the two industries compose the pillar of the modern industrial chain. The inseparable industrial connection determines that the development of producer services inevitably benefits local manufacturing industries by providing sufficient professional support to the latter. As is proved by many studies [14,15], through deepening the specialized production and lowering searching and transaction costs, producer services promote the resource allocation efficiency among production departments and thus impel the alleviation of the industrial chain. Moreover, due to the continuous refinement of professional division of production contemporarily, the value-added parts of the industrial chain continue to extend to the upper and lower ends where most of the works are accomplished by the sectors of producer services, forcing modern manufacturing to integrate with the producer service industry for the improvement of technology, production and factor allocation efficiency [16]. Especially in the highly technological and innovative phase of industry 4.0 where the increasingly complex information and communication of artificial intelligence systems require a more professional service for the new man–machine interface modes [17]. Therefore, the integration of manufacturing with producer services is expected to become the chance of a second take-off for manufacturing [18].

Since China joined the World Trade Organization (WTO) in the year of 2001, the volume of Chinese export manufactured products has been rising continuously, reaching to USD 2352.021 billion in 2018 from USD 402.1 billion in 2000, with a rapid average annual growth rate of 10.95%. However, even though China has become a dominant manufacturing exporter, it is nonetheless a strong exporter in terms of the role in the international division of the global value chain, as most Chinese export enterprises produce labor-intensive goods with simple techniques and low added values, making full use of the comparative advantages of low-cost labor in international processing trade [19]. According to Lemoine and $\ddot{U}$nal-Kesenci (2007) [20], the export structure of China accentuated the shortage of highly-skilled personnel. Koopman et al. (2012) [21] also found that among Chinese manufactured exports, the domestic value added for technological sophisticated sectors was particularly low, with the share merely less than 30%. In recent years, China began to take measures such as the implementation of the “Supply-side Reform” to upgrade manufacturing system and optimize the export structure as domestic labor cost gradually increased, and the technological level of Chinese export manufactured products has been heightened significantly according to the export data. Nevertheless, China is still obviously lagging behind the developed countries in the competition of manufacturing technology and resource utilization, one reason for which, as Yang and Zhang (2008) [22] held, is that the weak support from domestic producer services for manufacturing has led to the low-end position on global value chain where most of Chinese export producers are embedded in for the long term. In comparison, the results of existent studies on regional differences of manufacturing efficiency and profitability in China by domestic scholars show that in regions where the development of producer services is relatively more mature, such as the city groups in the Yangtze River Delta [23] and the Pearl River Delta [24], local manufacturing is much more advanced than that of other places. Therefore, a comprehensive research into the influence of producer services on export manufacturing and the regional heterogeneity in China is particularly urgent in order to gain an overall grasp of the Chinese export industrial chain in the complex context of Chinese regional economic imbalances.

Currently some progresses have been made in research on producer services agglomeration and its influence on manufacturing export. However, there are still some common problems: (1) because of the different indexes chosen to appraise producer services agglomeration and manufacturing productivity, specific results on particular problems can be varied among studies; (2) there still lacks specific exploration of cases of big developing countries like China, particularly in terms of the regional heterogeneity, as it is known that manifest gaps between regional economies usually exist in these countries; (3) suitable industrial policy suggestions for certain areas taking distinct local economic conditions, industrial bases and institutional environments into consideration. The contribution of this paper is that, using the two well-accepted indexes, i.e., local entropy and export technological complexity, this study aims to check the overall and spatial performances of urban producer services clusters effecting the techniques of local export manufacturing in cities of the largest developing country, China, to obtain reasonable outcomes and put forward suitable industrial policy suggestions. The remainder of this study is organized as follows. Section 2 reviews the existent literature on related issues. Section 3 introduces the measurement model and data base. Section 4 illustrates the spatial distribution of urban producer services agglomeration and techniques of exporting manufactured products through figures. Section 5 describes the analysis of empirical results. Section 6 makes conclusions and discussions for the study.

## 2. Literature Review

Existing studies have revealed that the spatial patterns of producer services clusters remained consistent with the revolution of urbanization, suburbanization and gentrification in recent decades: in the middle of the last century, producer service enterprises were concentrated to the CBDs of metropolises [25]; during the 1970s, with the rise of suburbanization, large quantity of producer service enterprises started to move to the suburbs [26]; since the 1980s, the pattern of producer services agglomeration in the suburbs has been further consolidated, establishing the spatial functional division with the central urban areas [27]; however, Coffey et al. (2005) [28] discovered that along with the revitalization of the city centers and to satisfy the requirement for an information-rich and contact-intensive environment of business, high-end producer services tend to be located in the CBDs. Besides, scholars have also noticed the influence of the remarkable progress of information technology on producer services agglomeration. Even though producer service enterprises are endowed with greater freedom in location selection, which may reduce the degree of agglomeration [15], the reinforcement of the technological level of the industry amplifies the invisible “iceberg cost” such as information dissipation and transaction cost caused by distance at the same time, therefore industrial agglomeration is still prevailing for knowledge-intensive producer services [4], and generates an effect of spinout amongst enterprises that continue to enlarge the scale of the agglomeration [9].

Existing studies on the relationship between producer services and manufacturing mainly focus on coordination analysis, mechanism analysis, the value chain, etc. The empirical research of Macpherson (2008) [29] revealed that the use of external productive services successfully enhanced technology progress and product innovation of manufacturing enterprises between the years 1994 and 2005 in New York. Eswaran and Kotwal (2002) [30] advocated that the technology spillover effects of producer services agglomeration significantly affect the labor productivity level of manufacturing. Simmie and Strambach (2006) [31] proposed that the agglomeration of knowledge-intensive service industries is conducive to the refinement of knowledge division and the promotion of technique level of manufacturing. In a new scenario, Di Nardo et al. (2015) developed highly intelligent and innovative human reliability analysis methods to quantify the error probability of human behavior and emphasized necessary training of human–machine interaction skills to workers [32], which should be accomplished by the integration of both producer service industry and manufacturing.

On the link between producer services and export, many studies subscribed to the enhancement of export from the support of producer services. Using data between 1994 and 2004 of OECD countries, Francois and Wörz (2008) [33] empirically proved that producer services can significantly promote the export competitiveness of downstream industries. Constantinescu et al. (2017) [34] emphasized that under the background of international division of production, close interaction with producer services enables export industries to achieve greater advantages on both the domestic and international markets. Some scholars further investigated the effect of producer services on export technology. Conducting research on the case of OECD countries, Castejón et al. (2008) [1] verified that through the interaction with the producer service industry, the technique of export manufacturing products kept improving sustainably. Zhang et al. (2016) [35] suggested that the agglomeration of producer services contributes to the export upgrade mainly through the reinforcement of R&D services and intellectual support.

On the basis of previous studies, this study tries to test the following two hypotheses: (1) the expansion of urban producer service agglomeration enhances the technological complexity of export manufactured products in Chinese cities; (2) the performance of producer service agglomeration affecting the technique level of export manufacturing varies in the different city locations. Once the hypotheses are proved to be valid, appropriate regional industrial policy suggestions will be raised based on the findings.

## 3. Measurement Model and Data Processing

Hausmann et al. (2005) [36] designed the index of export technology complexity widely used in academia to measure the level of technical content of export products and Xu and Lu (2009) [37] amended the index. This paper uses the amended index to evaluate the export technology complexity of manufactured products of each city. Furthermore, utilizing location entropy as the measurement of the agglomeration of urban producer service industry, we systematically investigate the support of producer service system for local export manufacturing during the period 2000–2015 in Chinese prefecture cities mainly through regressing the location entropy with the technology complexity index of export manufactured products in various ways of panel data regression after a visualization analysis of the two indexes. Meanwhile, to explore the spatial characteristics of the connection between the two industries, we classify samples into four groups according to Chinese economic geography, namely the east, the center, the west and the northeast regions, and then, respectively, conduct regressions for each group.

### 3.1. Measurement of Producer Service Industry Agglomeration (PSIA)

With reference to the latest classification of the producer service industry by National Bureau of Statistics of China and taking into account data availability, this study selects the transportation industry, wholesale and retail industry, finance and insurance industry, computer and information service industry, scientific research service industry, and leasing and business service industry as components of the producer service industry. Using annual employment data in each city from China City Statistical Yearbooks (2001−2016), we first sum the annual employment population of the six industries above by cities to get the total labor value of producer service industry in each city every year, and then calculate the annual location entropy of the producer service industry of each city as follows:

(1) $$E_{ij}=\;\frac{L_{ij}/\sum_{j}^{m}L_{ij}}{\sum_{i}^{n}L_{ij}/\sum_{i}^{n}\sum_{j}^{m}L_{ij}}$$

In Equation (1), $L_{ij}$ represents the labor forces scale of the *j*–th industry, namely the producer service industry, in the *i*–th city; *n* is the number of cities and *m* is the types of industries. It should be emphasized that in the calculation of location entropy, producer service industry is summed as a whole rather than divided into six branches. Location entropy is widely used to measure the level of regional industrial agglomeration, where a value greater than one suggests that local industry has a comparative advantage [38]. Thence we use location entropy value $E_{ij}$ to indicate the maturity and competitiveness of producer service industry in the *i*–th city.

### 3.2. Measurement of Export Technology Complexity of Manufactured Products (ETCMP)

Export technology complexity is an index used to measure the quality of export goods, compare the procedural level of different countries, and explore the law of trade and income. Taking the revealed comparative advantage of export products in targeted countries as the weights, Hausmann et al. (2005) [36] used the weighted average of per capita GDP (PGDP) to calculate the index of technical complexity of each kind of export commodity. Hereafter, national export complexity is obtained by weighted sum of the standardized indexes of various commodities with export shares as the weights. On the one hand, this method is based on the premise that the country with higher income possesses the more sophisticated technology using PGDP as the weight; on the other hand, regional comparative advantages of different commodities are also used to adjust the weight, insuring the accuracy of the index in different regions. Replacing export and income data with provincial-level ones, Xu and Lu (2009) [37] applied the method to research on Chinese provincial export technology complexity. Referring to the previous studies, we select export data of manufactured goods during the period 2000–2015 from China Customs Import and Export Database, unify the first 6 digits of HS codes of each year according to the HS07 edition, and gain the index of ETCMP on prefecture level as follows:

First we calculate the technical complexity of the *j*–th commodity (TCC_j_)

(2) $${\mathrm{TCC}}_{j}\;=\;\sum_{i}\frac{q_{\mathrm{ij}}/Q_{i}}{\sum_{i}\left(q_{\mathrm{ij}}/Q_{i}\right)}{\mathrm{PGDP}}_{i}$$

where $q_{\mathrm{ij}}$ is the export quantity of the *j*–th commodity in the *i*-th city, $Q_{i}$ the total export volume of the *i*-th city, and ${\mathrm{PGDP}}_{i}$ per capita GDP of the *i*-th city. The coefficient $\frac{q_{\mathrm{ij}}/Q_{i}}{\sum_{i}\left(q_{\mathrm{ij}}/Q_{i}\right)}$ demonstrates the revealed comparative advantage of the export of the *j*–th commodity in the *i*-th city. In case of deviation from the real situation caused by goods of low quality and with the reference to related study [37], we adjust TCC_j_ with the coefficient c_ij_ using price per unit of goods as the measurement of quality:

(3) $${c_{\mathrm{ij}}}_{\;}=\;\frac{{\mathrm{price}}_{\mathrm{ij}}}{\sum_{i}\left(\mu_{\mathrm{ij}}{*\mathrm{price}}_{\mathrm{ij}}\right)}$$

In Equation (3), ${\mathrm{price}}_{\mathrm{ij}}$ is the export price per unit of the *j*–th commodity in the *i*-th city, $\mu_{\mathrm{ij}}$ the proportion of export volume of the *j*–th commodity in the *i*-th city. Thus we get the new form of TCC_j_

(4) $$TCC_{j}^{new}={\left(C_{\mathrm{ij}}\;\right)}^{\lambda }*TCC_{j}$$

where λ = 0.2 according to Xu (2007) [39]. Then the revised index ${\mathrm{TCC}}_{j}^{\mathrm{new}}$ can be added to the prefecture level to obtain the ETCMP of each city:

(5) $${\mathrm{ETCMP}}_{i}\;=\;\sum_{j}(\frac{q_{\mathrm{ij}}}{Q_{i}})*TCC_{j}^{new}$$

### 3.3. Selection of Samples

Among the current 293 prefecture cities over the Chinese mainland, 283 of them along with four municipalities were selected as samples in this study. Six cities in Tibet Autonomous Region, two in Xinjiang Autonomous Region and two in Hainan Province were deleted due to seriously missing data. Prefecture-level administrative divisions of ethnic minority in eleven provinces and autonomous regions, such as autonomous prefectures, areas, and leagues, were also not included because of their special economic-social environment, limited varieties of manufactured export goods and the missing urban data. Although the former prefecture Laiwu of Shandong Province was merged into the provincial capital, Jinan, in 2019, it existed as a prefecture city all through the period under study. Therefore, it was still counted as a prefecture city in this study. So in the total sample, the number of prefecture cities was 284 and the total number of cities was 288.

### 3.4. Modeling and Data Processing

To verify the effects of PSIA on ETCMP in cities across the country, with indexes of ETCMP and PSIA, respectively, as the explained and explanatory variable, several empirical models of panel data were constructed, i.e., the pooled ordinary least squares model (OLS), the individual fixed effect model (FE), the individual and time fixed effect model (FE_TM) and the random effect model (RE). To reduce endogenous problems caused by the correlation among various elements in the operation of the economic system, this study also adopted the dynamic panel system GMM model (sys_GMM) which effectively ensured the robustness of the estimation results. Except for OLS models, other models were all vce robust regressions.

Economic development level, investment structure, economic opening degree, R&D intensity, industrial structure as well as urban population scale were involved in the model as control variables, respectively, represented by the data of per capita GDP (PGDP), fixed asset investment (FAI), foreign direct investment (FDI), proportion of fiscal R&D investment to GDP (R&D), proportion of tertiary industry to GDP (tertiary) and population density (popdens) from China City Statistical Yearbooks (2001-2016). The first three control variables are the logarithms and are divided by relative deflators: PGDP by CPI, FAI and FDI by the Fixed Asset Investment Price Index. Furthermore, FDI data were also processed with the annual average USD to RMB exchange rates.

The sys_GMM model incorporates the lag period values of the explained variable into the regression as explanatory variables. In this study, we added the first and second order lags of the explained variable L1(ETCMP) and L2(ETCMP) to examine the effect of the historical performance of export manufacturing itself.

In addition to the full-sample regression, this study also specifically analyzes the regional effects of the eastern, central, western and northeastern parts of China by adding regional dummy variables *east, center, west and ne* into the model according to the location of each city.

## 4. Spatiotemporal Evolution Characteristics of PSIA and ETCMP

**Figure 1:**
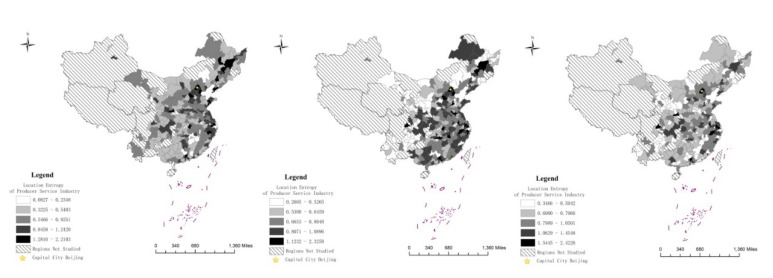
Urban Producer Service Industry Agglomeration (PSIA) in 2000, 2007 and 2015.

**Figure 2:**
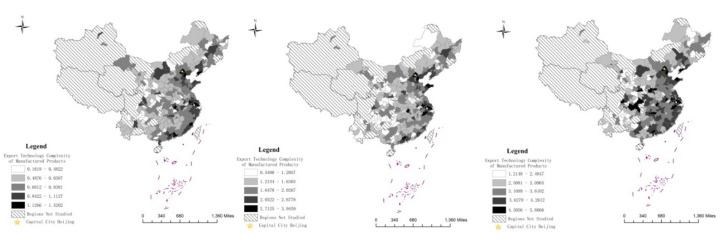
Urban Export Technology Complexity of Manufactured Products (ETCMP) in 2000, 2007 and 2015.

Based on the evaluation methodology mentioned above, this paper identifies PSIA and ETCMP through Stata 14 software. In order to reveal the longitudinal and spatial characteristics of the statistics, this paper presents a visualization analysis using data from 2000, 2007 and 2015, respectively, based on ArcGIS. Figure 1 and Figure 2 clarify the spatial differentiation of PSIA and ETCMP, respectively.

As is displayed in Figure 1, the agglomeration scale of China’s urban PSIA continued to increase during the study period, with the value range rising from 0.0827−2.2103 in 2000 to 0.3466−2.4226 in 2015. From the perspective of spatial distribution, it was evitable that developed cities in the Yangtze River Delta and the Pearl River Delta, as well as the capital city Beijing, always had the highest PSIA level throughout the study period, and the advantage has been expanded in the later period as the agglomeration of cities in the highest group was obviously narrowed down to merely these areas in 2015. In contrast, the figure shows that in 2000 and 2007, PSIA was distributed rather evenly: it was common to see cities of the highest group appear in other areas. This confirms the strengthening agglomeration tendency of the producer service industry to cities with a strong economic basis and favorable industrial environment, even though the industry also continuously develops all over the country. Likewise, on the regional level there also appeared the phenomenon of the producer service industry being concentrated to regional center cities, especially in the central area and the Beijing–Tianjin–Hebei areas where the indexes of PSIA rose fast in provincial capital cities like Wuhan, Changsha, Hefei, Shijiazhuang, and the municipality Tianjin. Meanwhile, Compared to situations in 2000 and 2007, it is very clear that the agglomeration of the producer service industry in the west and northeast gradually lagged behind other regions in recent years, as most of cities there belonged the two lowest groups in 2015. However, it is worth noticing that the double economic centers of the southwestern region, Chengdu and Chongqing, kept their strong attractions to producer service enterprises even after 2007, maintaining the positions in the highest two groups in 2015, which was rare for the majority of western cities besides those very few newly emerging ones like Xi’an and Guiyang.

Figure 2 illustrates the evolutionary trend of ETCMP in Chinese prefecture cities during the period 2000−2015. Statistics show that ETCMP of Chinese cities also enhanced significantly from 0.1619−1.5202 in 2000 to 1.2148−5.8066 in 2015, especially for the modern manufacturing base and developed cities with powerful technological and innovative capacity. Similar to PSIA, the Yangtze River Delta, the Pearl River Delta and Beijing–Tianjin–Hebei area had constantly ranked in the highest group. In addition, domestic manufacturing powers like Shandong Peninsula City Group, and the West Coast Economic Zone of the Taiwan Straits in Fujian Province also manifested local technique ascendancy in export manufacturing. All of the regions above belong to the east region of China, demonstrating the dominant role in the manufacturing industry of the developed coastal area. Meanwhile, thanks to the consummate manufacturing foundation, and particularly the industrial transfer from eastern areas, cities of the central provinces of Henan, Anhui, Hubei, Hunan had also made remarkable progress in the improvement of ETCMP in recent years, and together with the neighboring eastern area had gradually formed a contiguous and competitive area of higher ETCMP. At the same time, as the important industrial and technology R&D base of western China, Chengdu and Chongqing also saw an obvious raise of ETCMP, and consequently boosted ETCMP of nearby cities, consolidating regional industrial and technological level of the Chengdu–Chongqing Economic Circle. Appearing in the medium group twice in 2000 and 2007, Chongqing made a particularly outstanding advancement in the promotion of technical content of export manufacturing, ascending to the highest group in 2015. However, many areas are always inferior in the field of export trade and technology innovation, and most of the cities in the lowest groups are located in the northwest region. Furthermore, as the old industrial area of China, many enterprises in the northeast area still follow the traditional production and management models and are stuck in this predicament; thus, most cities in this area were also included in the two lowest groups.

Analysis above establishes a grasp of the longitudinal and spatial characteristics of PSIA and ETCMP in sample cities. Based on this, we will proceed to the empirical analysis next to examine the influence of PSIA on ETCMP in Chinese cities, as well as whether heterogeneity exists.

## 5. Analysis of Empirical Results

### 5.1. Analysis of Results on the Full-Sample Level

A quantitative analysis on the full-sample panel data is carried out through OLS, FE, FE_TM, RE and sys_GMM models with the software of Stata 14. The empirical results of regressions are listed in Table 1. The row of “con” displays the estimated values of constants.

Based on the empirical results shown in Table 1, the estimated coefficients keep consistent on the whole, verifying the robustness of the model setting. Before regression of the FE model, this study also conducts a pooled OLS regression with an individual dummy variable and individual fixed effect regression that is not vce robust in order to test the appropriateness of the FE model. Due to the layout limit, detailed results of pooled OLS regression with individual dummy variables, and individual fixed effect regression that is not vce robust are omitted, and only reported briefly as follows: statistics of F test and most of the individual dummy variables are significant, which indicates a rejection of the null hypothesis that “all individual dummy variables are 0”. Therefore, it is justified that there exists an individual effect, and an individual fixed effect model is more appropriate than mixed regression. FE_TM model adds time effect into FE model and the result of the F test strongly rejects the null hypothesis of “no time effect”, confirming that time effect should be included in the model. The Breusch and Pagan LM test comparing RE model with pooled OLS model also suggests that random effect regression is better than mixed OLS regression, while the further Sargan–Hansen statistic rejects random effect. Thus the FE_TM model is the best among the previous four models.

As is mentioned above, sys_GMM regression is also carried out in case of variable endogeneity, absorbing the first and second lag terms of the explained variable into the model. Arellano–Bond statistic passes the first-order difference error correlation test, and the Sargan test of over identifying restrictions accepts the assumption that the selected instrumental variables are not related to the error term at the significance level of 10%, so the model setting is effective. Thus, according to the outcomes of serials of statistical tests, we will focus on the analysis of results of FE_TM and sys_GMM models below.

It can be seen that both the FE_TM and sys_GMM regressions reveal that on the full-sample level, the location entropy of the urban producer service industry is significantly and positively correlated with ETCMP, which indicates that the expansion of the producer service industry agglomeration generates a driving force for the improvement of ETCMP level through the mechanisms of deepening industrial division, strengthening knowledge spillover, and accelerating value flow on the industrial chain as is presumed in Hypothesis (1).

As with the control variables, the estimated coefficients of variable “PGDP” are significantly positive in both the FE_TM and sys_GMM regressions, suggesting that the level of urban economic development has a positive relationship with the ETCMP. The estimated coefficient of variable “FAI” is positive in both models, indicating that fixed asset investment promotes the manufacturing industry by offering high quality industrial facilities and infrastructures to manufacturers. However, the estimated coefficient is significant in the FE model but fails to pass the significance test in the sys_GMM regression, implying that as an infrastructure giant, the scale of construction over the country may be so large that it will surpass the level optimization, leading to a diminishing return of scale to the manufacturing sector, especially in the aspect of updated production techniques. It is unexpected that the results of variable “FDI” are insignificant statistically in both models, and the estimated coefficient in sys_GMM regression is even negative, contrary to the perdition that an open economic environment is conducive to technological improvement. A possible explanation may be that foreign investments mainly target labor-intensive processing sectors in the developed countries, profiting mostly from cheap labors rather than manufacturing technique improvement, thereby strengthening the role of low-end manufacturing of investees on the global industrial chain and rarely generating noticeable technical innovation locally. The estimated outcomes of variables “R&D” and “tertiary” are all positive but only significant in one type of regression, denoting that R&D input and industrial structure alleviation has begun to show enhancement of technical improvement in export manufacturing, although this is sometimes not stable. The urban population scale represented by the variable “popdens” has no significant effect on ETCMP. In addition, in sys_GMM model estimation, the estimated coefficients of the first and second-order lagging terms of the explained variable are significantly positive, indicating that there exists a path dependence effect in the technique development of urban export manufacturing in China, with the ETCMP in the previous periods having a positive impact on that of the current period.

### 5.2. Analysis of Results on the Regional Level

Quantitative analyses on regional level of the eastern, central, western and northeastern areas of China are individually conducted by the software of Stata 14, with regional dummy variables identifying the location of cities. Taking into account the statistical structure and variable endogeneity, regressions for all the four groups are in the form of the sys_GMM model. The empirical results of regressions are listed in Table 2. The row of “con” displays the estimated values of constants.

Based on the regression results displayed in Table 2, Arellano–Bond statistics for the lag terms of the explained variable in four groups all pass the first-order difference error correlation tests. The results of the Sargan test also accept the null hypothesis at the significance level of 10%, proving that an over identification problem does not exist. The estimated results validate Hypothesis (2): although the coefficients of PSIA of cities of all groups are positive, only those of the eastern and the central groups pass the statistical significance test. Such an outcome confirms that the reinforcement of PSIA has an evidently positive impact on the promotion of ETCMP in cities where the industrial structure is more advanced and complete. The reason why the estimated coefficient for the northeast part is statistically insignificant may be that the relatively limited sample size compared to other groups influences the statistical significance of regression—there are only three provinces and 34 prefecture cities in total in the northeast region. Apart from the statistical reason, the imperfect industrial structure in the west and the northeast of China should be mainly responsible for the different results from the east and center. As China’s important old industrial base, the regional mainstay industry of the northeast area has always been heavy industries which wane as the new economy emerges. For the west region, the majority is still in the primary phase of industrialization. Therefore, the scale and sophistication of producer service industry and high-end manufacturing in these two regions are relatively backward, causing the statistically insignificant result of the estimation.

The estimated coefficients of the first and second-order lagging terms of the explained variable are statistically significant and positive in all the four regions, verifying that the path dependence effect in the development of urban export manufacturing technology operates universally around Chinese cities, without being influenced by regional heterogeneity. However, except for the group of the central area, the estimated coefficients of regional dummy variables in the rest three groups all pass the significant tests, suggesting that the specific regional economic condition influenced by the city’s location does have a considerable impact on local ETCMP. As is displayed in the table, the estimated coefficient of variable “east” is significantly positive, signifying that the location in the east part of China endows cities with technical superiority in the export manufacturing industry, which can be attributed to the implementation of an unbalanced regional development strategy since the reform and expansion. For decades the regional strategy has been preferential to the coastal areas convenient for export processing trade, and thus the eastern area has become the growth pole and core of national economy, attracting and accumulating abundant material, human, technological and social capitals. Consequently there has formed an inherent advantage of eastern cities in industrial development and technological competition. By contrast, other regions are in an inferior position in the regional competition of technology innovation, industrial upgrade and economy growth, manifested by the coefficient of variable “center” being statistically insignificant, and those of variables “west” and “ne” being significantly negative. Such results indicate that the location has not brought about an obvious impact on the ETCMP yet in central cities as they have already begun to catch up with the eastern neighbors with regional industrial transfer and technological spillover in recent years; while for the rest groups, with frail industrial bases and insufficient infrastructure in the west, and the lagging industrial structure in the northeast, the speed of economic development and technological progress is slower in these two regions. Moreover, the disadvantaged locations far away from the developed east of the two regions also weaken the local ability to attract investment and technical personnel, causing a serious impediment to the rise in the technical content of export manufactured goods for local cities.

The estimated results of control variables generally keep consistent with those of the estimation on the full-sample level, showing that the mechanism of certain economic and social factors, like GDP per capita, FDI, industrial advanced degree and population density, affects the technical sophistication of export manufactured products in a similar way countrywide. Meanwhile, subtle differences are also worthy of note. Firstly, the statistical significance of the estimated coefficient of variable “FAI” varies according to the city’s location, being significant in the central and western areas where infrastructure basis is relatively weak, while it is insignificant in the eastern and northeastern regions with more complete infrastructures, which proves that recent major construction programs in the center and the west to tackle infrastructure shortcomings did make an important contribution to the improvement of the regional manufacturing technology level locally. Second, none of the estimated results of variable “R&D” are statistically significant for all groups, implying that the inadequate R&D input is still a common obstacle for regional manufacturing technique promotion, even in the developed east.

## 6. Conclusions and Discussion

Taking 284 Chinese prefecture cities (one former and 283 current) and 4 municipalities as samples, this paper examined PSI and ETCMP by calculating the local entropy of the producer service industry and the complexity index of export manufactured goods of each city during the period 2000–2015, and then conducted a spatial visualization analysis. Based on this, we also performed an empirical analysis with several panel data models using Stata 14 on the full-sample and regional levels, in order to check the support for the export manufacturing from the urban producer service industry, and the related regional differentiation. The results are as follows: (1) the producer service industry continued to concentrate in developed cities during the study period. On the national level, Beijing and cities of the Yangtze River and Pearl River Deltas enjoyed the highest PSIA level for the entire period. Regional economic center cities of the fast-developing areas in the central and southwestern China also manifested strong attraction to producer service industry, while the producer service industry in most parts of the west and the northeast gradually lagged behind, except for few newly emerging cities like Xi’an and Guiyang. (2) ETCMP of Chinese cities was also enhanced significantly, especially for the eastern coastal regions, and due to the regional industrial transfer, ETCMP in central areas neighboring the east, and the Chengdu–Chongqing Economic Circle in the southwest also promoted rapidly after 2007. However, ETCMP values were commonly lower than the average in most parts of the northwest and the northeast regions. (3) In terms of the empirical analysis, on the full-sample level the location entropy of the urban producer service industry is significantly and positively correlated with ETCMP, revealing that the expansion of PSIA in Chinese cities yields the power to boost urban ETCMP. (4) On the regional level, the reinforcement of PSIA has an evidently positive impact on the promotion of ETCMP for the eastern and the central cities, while due to regional economic differentiation, the estimated coefficients for the northeast and the west regions are statistically insignificant. For the regression results of the regional dummy variables, that of the eastern city group is significantly positive, while that of the central city group is statistically insignificant, and those of the western and the northeastern city groups are significantly negative, which confirms that there does exist regional heterogeneity in the technical development of the export manufacturing industry, and the influence on it from PSIA among Chinese cities.

Based on the location entropy and export technology complexity, which have been accepted as reasonable evaluations for industrial agglomeration and export technical content around academia, the result of spatial visualization analysis shows that the producer service industry and export manufacturing technology have developed remarkably countrywide during the study period. Yet, unlike the improvement of ETCMP, which displayed undoubted trickle-down effect, the regional differentiation of PSIA has apparently expanded. Under the cumulative effect of agglomeration, producer service enterprises remain concentrated to the most economically-developed cities in the coastal eastern area like Beijing, Shanghai, Guangzhou and Shenzhen, in pursuit of the latest information, richest human capital and broadest market. Meanwhile, regional center cities in the fast developing central and the southwestern areas, such as Wuhan, Chengdu and Chongqing also began to demonstrate strong attraction to the producer service industry in the latter half of the study period. However, contrary to the Yangtze River and the Pearl River Deltas where producer services commonly flourished in many of the local cities, the rapid expansion of industrial cluster in the regional center cities of the central area and the Chengdu–Chongqing Economic Circle inevitably weakened the agglomeration capacity of producer services in nearby small and medium sized cities, owing to the limited scale of local markets, as is shown in Figure 1 where the ranks of many central and southwestern cities dropped sharply from 2007 to 2015. In other words, polarization of PSIA is taking place both at the national and the regional levels. Such results not only confirm the finding of previous studies that producer services increasingly agglomerate, but also seem to partly give an answer to the uncertain effect of information, communication and transportation technology revolution on the location selection of the producer service industry: as the Internet, high-speed rail and so on largely extends the spatial coverage of producer services, it allows producer service enterprises to agglomerate to big center cities with greater material, technology and human capital more freely without losing clients in nearby small and medium sized cities.

The results of empirical analysis acknowledge the coordinated relationship between PSIA and ETCMP amongst Chinese cities as there exists the significant and positive relationship between the two variables. Examining by areas, it is also evident that with powerful support of local producer service industry, export manufacturing in the eastern and central areas has undergone a continuous technology upgrade, while since a great many cities in the west and the northeast lack favorable markets and institutional environments for the producer service industry to develop, the enhancement of PSIA to ETCMP there is quite vague. Such a result confirms the prevalent conclusions in existent studies on Chinese regional economics where the producer service industry is more developed, and where the manufacturing is more advanced. Moreover, except for the regional gaps among the eastern, central, western and northeastern areas, which have been widely recognized, it is also noteworthy that differentiation between the north and the south also gradually emerges, as the development of producer services and high-end export manufacturing industries in the northwest and the northeast keep falling behind continuously, and the inter-industrial enhancement remains insignificant, which should raise the attention in future studies. Besides, regional dummy variables also have a different significant effect on ETCMP, validating the influence on the export manufacturing development from city locations. Therefore, industrial polices suitable for local economic and industrial conditions, and appropriate adjustment to local economic institutions is a must for prefecture governments.

According to the outcomes, several implications and strategies can be made: first, it is suggested to deepen the integration of manufacturing with the producer service industry which should be an effective way to optimize the export structure, and alleviate the technical content and added value of export finished products; second, since the central area has set an example for regional industrial upgrade, local governments of economically backward areas like the northwest and the northeast should take measures to attract modern manufacturing and service industrial transfer, so as to promote institutional environment, accelerate infrastructure renovation and change the traditional production mode. Third, R&D activities and technological innovation should be greatly encouraged.

With restrictions of data availability, this paper studies the issue of updating Chinese urban producer service agglomeration and export manufacturing on a relatively broad level of the prefecture. Nevertheless, in order to refine the research on urban industrial coordination and upgrade, further studies should conquer this limitation, exploring the application of big data, cloud computing, Internet of Things, python technology and so on to widen data resources and deepen the research dimension to the county and district level, even to the town and street level, so that the theme of urban industrial development can be clarified from multiple perspectives like urban functional zoning, urban industrial ecosystem, urbanization, etc.

In short, with the contemporary industrial transition, it is necessary to recognize the complexity of urban export manufacturing technology from the perspective of industrial connection and agglomeration. Furthermore, the scientific selection of evaluative indexes and reasonable establishment of analysis structure including evolutionary dynamics visualization and comparative group regressions are of particular importance for studies on countries with distinct regional heterogeneity, which is expected to generate a guide to the amelioration of regional industrial policy. In the future, a main direction for research should be the establishment of a structural model for Chinese urban industrial chain upgrade with more refined data.

## Figures and Tables

**Table 1:** Full-Sample Empirical Results of Effects of PSIA on ETCMP.

Variables/Models	OLS	FE	FE_TM	RE	sys_GMM
PSIA	0.2831 (0.85)	0.0603 *** (3.08)	0.0394 *** (3.16)	0.0299 *** (3.28)	0.0561 * (1.75)
L1(METC)					0.5342 *** (9.93)
L2(METC)					0.1714 *** (4.61)
Per capita GDP (PGDP)	0.9739 *** (22.01)	1.2964 *** (10.31)	0.4796 *** (3.87)	1.1470 *** (15.87)	0.6371 *** (7.47)
Fixed asset investment (FAI)	0.3171 *** (13.70)	0.2237 *** (3.31)	0.0714 ** (2.12)	0.0307 *** (7.59)	0.0315 (1.13)
Foreign direct investment (FDI)	−0.0965 *** (−7.96)	−0.0106 (−0.67)	0.0001 (0.01)	−0.0811 *** (−7.37)	−0.0146 (−1.30)
Proportion of fiscal R&D investment to GDP (R&D)	0.0065 (1.01)	0.1474 *** (2.83)	0.0094 * (1.89)	0.0114 ** (2.14)	0.0080 (1.03)
Proportion of tertiary industry to GDP (tertiary)	0.0289 (0.13)	1.8145 *** (4.54)	0.0046 (0.02)	0.8373 *** (3.33)	1.2062 *** (2.73)
Population density (popdens)	−1.5765 *** (−2.88)	−0.2003 (−0.30)	−0.6575 (−1.54)	−1.9071 *** (−3.88)	−0.4474 (−0.90)
con	−1.9591 *** (−6.05)	−2.4186 ** (−2.42)	1.9103 *** (3.87)	−2.3692 *** (−4.19)	1.2668 ** (2.39)
R^2^	0.7718	0.7222	0.8227	0.7648	

*, **, *** indicate significance at the level of 10%, 5%, and 1%, respectively. Numbers in the brackets are t-values of regression coefficients.

**Table 2:** Empirical Results of Four Regional Groups.

Variables/Region	East	Center	West	Northeast
PSIA	0.6744 * (1.85)	0.0518 * (1.87)	0.0571 (0.72)	0.0437 (1.35)
L1(METC)	0.5287 *** (9.86)	0.5351 *** (10.08)	0.5305*** (10.54)	0.5335 *** (10.01)
L2(METC)	0.1721 *** (4.63)	0.1732 *** (4.68)	0.1718 *** (4.70)	0.1727 *** (4.74)
east	0.1622 * (1.75)			
center		0.3900 (0.49)		
west			−0.0824 * (−1.79)	
ne				−0.2224 * (−1.93)
PGDP	0.6525 *** (7.43)	0.6538 *** (7.15)	0.6538 *** (8.91)	0.6573 *** (8.15)
FAI	0.1999 (0.70)	0.0183 *** (3.62)	0.0236 *** (2.91)	0.0206 (0.80)
FDI	−0.0055 (−0.44)	−0.0111 (−0.92)	−0.0121 (−1.05)	−0.0171 (−1.38)
R&D	0.0069 (0.91)	0.0066 (0.92)	0.0076 (1.04)	0.0082 (1.04)
tertiary	1.1741 *** (2.71)	1.3274 *** (2.83)	1.1915 ** (2.49)	1.1737 *** (2.61)
popdens	0.1438 (0.22)	−0.2593 (−0.42)	−0.2729 (−0.51)	−0.5636 (−1.01)
con	1.3609 *** (2.60)	1.4036 *** (2.63)	1.3285 *** (2.61)	1.4651 *** (2.73)

*, **, *** indicate significance at the level of 10%, 5%, and 1% respectively. Numbers in the brackets are t-values of regression coefficients.
